# A Single Amino Acid Substitution at Residue 218 of Hemagglutinin Improves the Growth of Influenza A(H7N9) Candidate Vaccine Viruses

**DOI:** 10.1128/JVI.00570-19

**Published:** 2019-09-12

**Authors:** Xing Li, Yamei Gao, Zhiping Ye

**Affiliations:** a Division of Viral Products, Center for Biologics Evaluation and Research, Silver Spring, Maryland, USA; Icahn School of Medicine at Mount Sinai

**Keywords:** influenza A(H7N9) virus, balancing HA and NA functions, pandemic preparedness, pathogenesis in ferret, vaccine protein yield, virus replication

## Abstract

The potential avian influenza pandemic remains a threat to public health, as the avian-origin influenza A(H7N9) virus has caused more than 1,560 laboratory-confirmed human infections since 2013, with nearly 40% mortality. Development of low-pathogenic candidate vaccine viruses (CVVs) for vaccine production is essential for pandemic preparedness. However, the suboptimal growth of CVVs in mammalian cells and chicken eggs is often a challenge. By introducing a single adaptive substitution, G218E, into the hemagglutinin (HA), we generated reassortant A(H7N9)-G218E CVVs that were characterized by significantly enhanced growth in both cells and eggs. These G218E CVVs retained the original antigenicity, as determined by a hemagglutination inhibition assay, and effectively protected ferrets from lethal challenge with the highly pathogenic parental virus. We found that the suboptimal replication of the parental H7 CVVs was associated with impeded progeny virus release as a result of strong HA receptor binding relative to weak neuraminidase (NA) cleavage of receptors. In contrast, the G218E-mediated growth improvement was attributed to relatively balanced HA and NA functions, resulted from reduced HA binding to both human- and avian-type receptors, and thus facilitated NA-mediated virus release. Our findings revealed that a single amino acid mutation at residue 218 of the HA improved the growth of A(H7N9) influenza virus by balancing HA and NA functions, shedding light on an alternative approach for optimizing certain influenza CVVs.

**IMPORTANCE** The circulating avian influenza A(H7N9) has caused recurrent epidemic waves with high mortality in China since 2013, in which the alarming fifth wave crossing 2016 and 2017 was highlighted by a large number of human infections and the emergence of highly pathogenic avian influenza (HPAI) A(H7N9) strains in human cases. We generated low-pathogenic reassortant CVVs derived from the emerging A(H7N9) with improved virus replication and protein yield in both MDCK cells and eggs by introducing a single substitution, G218E, into HA, which was associated with reducing HA receptor binding and subsequently balancing HA-NA functions. The *in vitro* and *in vivo* experiments demonstrated comparable antigenicity of the G218E CVVs with that of their wild-type (WT) counterparts, and both the WT and the G218E CVVs fully protected ferrets from parental HPAI virus challenge. With high yield traits and the anticipated antigenicity, the G218E CVVs should benefit preparedness against the threat of an A(H7N9) influenza pandemic.

## Abstract (precis)

The circulating avian influenza A(H7N9) has caused recurrent epidemic waves with high mortality in China since 2013, in which the alarming fifth wave crossing 2016 and 2017 was highlighted by a large number of human infections and the emergence of highly pathogenic avian influenza (HPAI) A(H7N9) strains in human cases. We generated low-pathogenic reassortant CVVs derived from the emerging A(H7N9) with improved virus replication and protein yield in both MDCK cells and eggs by introducing a single substitution, G218E, into HA, which was associated with reducing HA receptor binding and subsequently balancing HA-NA functions. The *in vitro* and *in vivo* experiments demonstrated comparable antigenicity of the G218E CVVs with that of their wild-type (WT) counterparts, and both the WT and the G218E CVVs fully protected ferrets from parental HPAI virus challenge. With high yield traits and the anticipated antigenicity, the G218E CVVs should benefit preparedness against the threat of an A(H7N9) influenza pandemic.

## INTRODUCTION

Since its emergence in spring 2013, the avian-origin influenza A(H7N9) virus has caused six epidemic waves in China with a total of 1,567 laboratory-confirmed cases of human infection there, including at least 615 deaths as of 6 March 2019 (http://www.fao.org/ag/againfo/programmes/en/empres/H7N9/Situation_update.html). The fifth wave (October 2016 through September 2017) began earlier, spread broader, and caused more human infections than other waves (1). Moreover, highly pathogenic avian influenza (HPAI) A(H7N9) variants were isolated from 32 human cases as well as dozens of poultry and environmental samples, and caused 27 outbreaks in poultry farms in China from July 2016 to November 2018 (2). The human-isolated HPAI variant A(H7N9) evolved from the currently circulating low-pathogenic A(H7N9) lineage (3), can transmit among ferrets by respiratory droplets, and is more pathogenic to ferrets and mice than the low-pathogenic strain (4, 5). The dynamics of A(H7N9) prevalence have raised serious concerns about a potential pandemic and promoted global efforts for pandemic preparedness. At present, vaccination is the essential means to protect against influenza illness; therefore, selection and development of candidate vaccine viruses (CVVs) with high growth traits are the critical steps toward timely vaccine production. However, the suboptimal growth of avian CVVs in mammalian cells and chicken embryonated eggs (eggs) is often a challenge for pandemic vaccine production due to host range restriction.

As a part of the pandemic preparedness for the avian influenza A(H7N9), the World Health Organization (WHO) has recommended the prepandemic CVV strains, including A/Shanghai/2/2013 (SH2) and A/Guangdong/17SF003/2016 (GD17), based on the updated antigenic, genetic, and epidemiologic data. We generated reassortant CVVs rgSH2WT from SH2 and rgGD17 from GD17. In agreement with other reports (6, 7), the rgSH2 and rgGD17 reassortants showed a suboptimal growth phenotype in both eggs and mammalian cells, which could undermine the effective response to the potential A(H7N9) prevalence in the human population.

To meet the high vaccine demand and urgent vaccination timetable in case of a pandemic outbreak, scientists and the vaccine industry have been making great efforts to develop and improve the CVVs that grow well in eggs or MDCK cells, as well as retain the antigenicity. To date, several groups have reported the results of selecting egg-adapted A(H7N9) CVVs by serial passage in the egg allantoic cavity and identified a few amino acid substitutions associated with egg adaptation in hemagglutinin (HA), including G218E (8), N158D (6, 7, 9, 10), N132D (6, 9, 10), G198E (6, 7), and G205E (7) in H3 numbering. Despite of their locations in the putative antigenic sites, these HA mutations appear not to significantly change the antigenicity of the CVVs. Moreover, manipulation of the packaging signals of the neuraminidase (NA) gene achieves improved the growth of A(H7N9) CVV (8).

We had laid the ground work for generating adaptive H7 CVV by characterization of a cell-adapted strain from a H7N3 CVV, A/mallard/Netherlands/12/2000 (NL12), where we selected the cell-adaptive NL12 in Madin-Darby canine kidney (MDCK) cells and attributed the adaptive phenotype to a single amino acid substitution, G218E (H3 numbering), in HA. Based on the genetic similarity between the HAs of the A(H7N9) and NL12, we introduced the G218E substitution into the HAs of rgSH2 and rgGD17 to promote viral growth. Indeed, G218E substitution significantly improved rgSH2 and rgGD17 replication in both eggs and mammalian cells, which is consistent with the recently reported G218E high-growth phenotype in another A(H7N9) CVV, A/Anhui/1/2013 (8). Furthermore, we compared the immunogenicity of the G218E variants with that of the wild-type (WT) counterparts and investigated the mechanisms underlying G218E-associated high growth of A(H7N9) CVV in this study.

## RESULTS

### Selection of MDCK-adapted NL12 variant.

**FIG 1:**
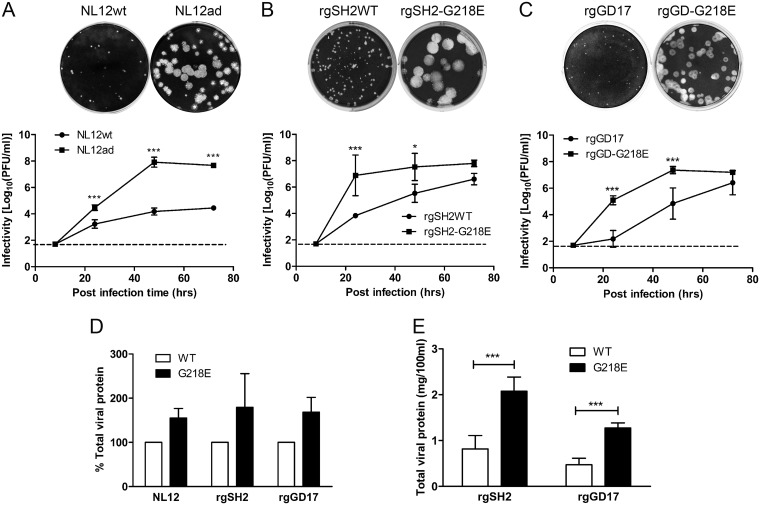
Growth characteristics of H7 viruses. (A to C) Comparison of plaque morphology and multistep growth curves (after infection with each virus at an MOI of 0.01) of NL12ad with NL12WT (A), rgSH2-G218E with rgSH2WT (B), and rgGD-G218E with rgGD17 (C). The dashed lines indicate the assay limit. (D) Relative total viral protein yield from purified MDCK-grown NL12ad, rgSH2-G218E, and rgGD-G218E to the wild-type counterparts, respectively. (E) Total viral protein yield of egg-grown virus per 100 ml of egg allantoic fluid. Each data set was from at least three independent experiments. *, *P* < 0.05; ***, *P* < 0.001 (two-way ANOVA with Bonferroni posttest).

The WT H7N3 virus NL12 (NL12WT) replicated efficiently in eggs but grew poorly in MDCK cells, with a low infectivity and tiny plaque formation. To select the cell-adapted strain, we consecutively passaged NL12WT (multiplicity of infection [MOI] of 0.01) in MDCK cells. After three passages, virus titer increased from 1:8 (after first passage) to 1:64, and large plaques were dominant in MDCK monolayers. The NL12ad was purified by two rounds of plaque pick of the large plaques, which replicated more competently in MDCK cells (Fig. 1A).

### Association of G218E in HA with MDCK adaptation and improved growth of H7 viruses.

Sequencing analysis of NL12ad revealed two amino acid substitutions in HA, G218E and K328R (H3 numbering). No mutation was identified in NA. To identify the substitution responsible for the MDCK adaptation, we generated 7:1 (H7N1) reassortants rgNL12WT, rgNL12-G218E, rgNL12-K328R, and rgNL12-G218E/K328R. The rgNL12-G218E formed large plaques with sizes similar to that formed by rgNL12-G218E/K328R, while the plaque size of rgNL12-K328R was similar to that formed by rgNL12WT (data not shown). In addition, the multiple-step growth curves of rgNL12-G218E and rgNL12-G218E/K328R were similar, and rgNL12-K328R exhibited growth kinetics similar to those of rgNL12WT (data not shown), indicating that G218E was the determinant substitution for the NL12ad phenotype.

Based on the genetic and antigenic similarity between the HAs of SH2 and NL12, we introduced G218E into the HA of the low-yield A(H7N9) CVV rgSH2. In agreement with the G218E phenotype in NL12, rgSH2-G218E significantly outgrew rgSH2WT in both MDCK cells and eggs. rgSH2-G218E formed large plaques and replicated more efficiently in MDCK cells (Fig. 1B). The total viral protein yield of rgSH2-G218E increased by 92.5% in MDCK cells and 154% in eggs compared to that of the wild-type counterpart (Fig. 1D and E).

The HPAI GD17 emerged in the fifth wave was evolved from the low-pathogenic A(H7N9) but reacted poorly to ferret antiserum induced by SH2. Aligned with SH2 HA, GD17 HA has 12 amino acid substitutions in addition to the insertion of the multibasic cleavage site, including S127N in antigenic site A, L226Q in the receptor-binding site (RBS) and antigenic site D (11), and A134V that was attributed to antigenic drift (12). Meanwhile, R292K, a mutation attributed to neuraminidase inhibitor (NAi) resistance (13), was identified in GD17 NA. Therefore, we generated the low-pathogenic A(H7N9) CVV rgGD17 from the HPAI GD17WT by removing the multibasic motif in HA and introducing K292R in NA. rgGD17 also grew poorly in both eggs and MDCK cells. Similarly, we introduced G218E into the HA of rgGD17 and achieved significantly improved virus growth in both MDCK cells (68.3% increase, Fig. 1C and D) and eggs (170% increase, Fig. 1E) compared to rgGD17.

### Antigenicity and protection of ferrets from HPAI H7N9 infection.

**FIG 2:**
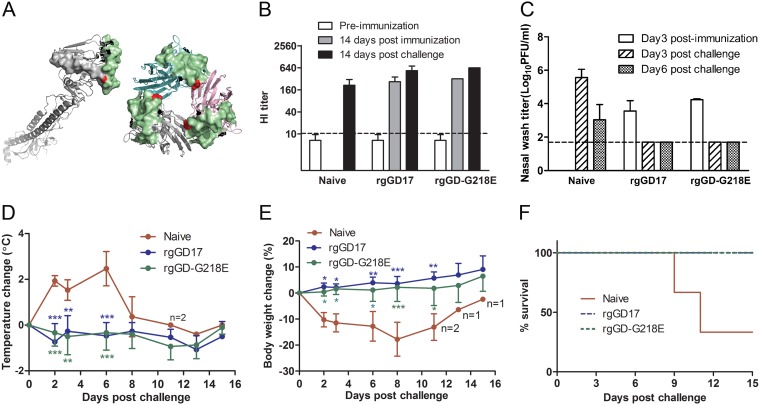
Protection of ferrets from HPAI GD17WT infection. (A) Location of G218E in H7 HA. The H7 HA structure was modeled by Swiss-Model with a H7 HA (PDB 4LN8) as the template, and three-dimensional images were created with the PyMOL Molecular Graphic System (https://pymol.org). (Left) Side view of the backbone of an H7 HA monomer; (right) top view of an H7 HA trimer. The green surface represents the RBS, the gray surface represents antigenic site D, and G218 is highlighted in red. (B) Geometric mean titers (GMT) of ferret serum HI antibody to GD17WT. An HI titer of <10 was assigned as 5 for GMT calculation. The dashed line indicates the assay limit. (C) Virus titers from nasal wash samples after rgGD17 or rgGD17-G218E immunization, followed by GD17WT challenge. The dashed line indicates the assay limit. (D) Postchallenge ferret body temperature change. (E) Percentage of ferret body weight change. The individual postchallenge body weight was normalized to that at day 0. Data are presented as means ± standard deviations (SD). *, *P* < 0.05; **, *P* < 0.01; ***, *P* < 0.001 (two-way ANOVA with Bonferroni posttest). (F) Percent ferret survival from lethal-dose GD17WT challenge.

As shown in Fig. 2A, the amino acid at position 218 of H7 HA is located in the putative antigenic site D (11). To examine the possible antigenic impact of G218E, we compared the antigenicity of G218E variants and the WT counterparts in ferrets. With the two pairs of ferret antisera elicited by the rgSH2WT/rgSH2-G218E and rgGD17/rgGD-G218E, the two-way hemagglutination inhibition (HI) assay results determined that the HI titers of each pair were within 2-fold difference (Table 1). Consistently, the microneutralization (MN) antibody titers of the rgGD17 antiserum and the rgGD-G218E antiserum were both within a 2-fold difference in neutralizing GD17WT, rgGD17, and rgGD-G218E (Table 1, values in parentheses), suggesting that the G218E substitution in rgSH2 HA or rgGD17 HA did not cause significant antigenic change. In addition, the HI antibodies elicited by both rgSH2 and rgGD17 showed certain cross-reactivities among the H7N9 strains, whereas rgGD17 and rgGD-G218E rather than rgSH2 induced MN antibodies that reacted well with both homologous and heterologous H7N9 viruses.

**TABLE 1:** Hemagglutination inhibition and microneutralization reactions of A(H7N9) reassortant viruses

Test antigen	Titer in ferret serum^*a*^			
	rgSH2WT	rgSH2-G218E	rgGD17	rgGD-G218E
rgSH2WT	320 (80)	320	320	640
rgSH2-G218E	320	320	160	640
rgGD17	80 (<10)	80	160 (80)	320 (226)
GD17WT^*b*^	160 (<10)	160	320 (40)	640 (160)
rgGD-G218E	160 (10)	160	320 (80)	640 (254)
NL12WT	80	80	40	40

a Each HI assay was performed with 1% horse red blood cells. MN titers are presented within parentheses (the geometric mean titer [GMT] of two to four repeats).

b MN assay with GD17WT was performed in a BSL-3 containment laboratory.

We further assessed the protection of the immune response to the rgGD17 and rgGD-G218E against GD17WT infection in a ferret model. Intranasal inoculation with live rgGD17 or rgGD-G218E in ferrets induced specific antibody response against both homologous virus and the parental HPAI GD17WT in 2 weeks, as confirmed by HI assay (Fig. 2B), while no obvious illness signs were observed (data not shown). To evaluate the effectiveness of the immune response elicited by rgGD17 and rgGD-G218E, the immunized ferrets were challenged with GD17WT virus. In the naive ferrets, GD17WT shedding lasted for at least 6 days (Fig. 2C), and all the three animals showed severe illness signs, including lethargy, lowered activity level, continuous fever (Fig. 2D), and progressive body weight loss (Fig. 2E) in the first week postchallenge. Two of the three ferrets were euthanized on days 9 and 11 postchallenge, respectively, due to severe illness (diarrhea, abnormal movements, and episodes of opisthodomos, as well as more than 20% body weight loss) (Fig. 2F). Necropsy of these two ferrets revealed extensive lesions in the lungs, but no live virus was found in samples from the tracheas, lungs, or spleens. Live virus was only detected in multiple areas of the brain from the ferret euthanized on day 9 postchallenge. The manifestations and course of the GD17WT infection in naive ferrets were similar to that reported for a HPAI H7N1 ferret model (14). In contrast, none of the rgGD17- or the rgGD-G218E-immunized ferrets showed obvious illness signs after GD17WT challenge. In addition, no virus shedding was detected from the immunized ferrets at day 3 or 6 postchallenge. These results demonstrated that both low-pathogenic reassortants rgGD-G218E and rgGD17 induced specific immune response that effectively protected the immunized ferrets from lethal-dose challenge of parental HPAI GD17WT.

### Reduced binding of G218E variants to MDCK cells.

**FIG 3:**
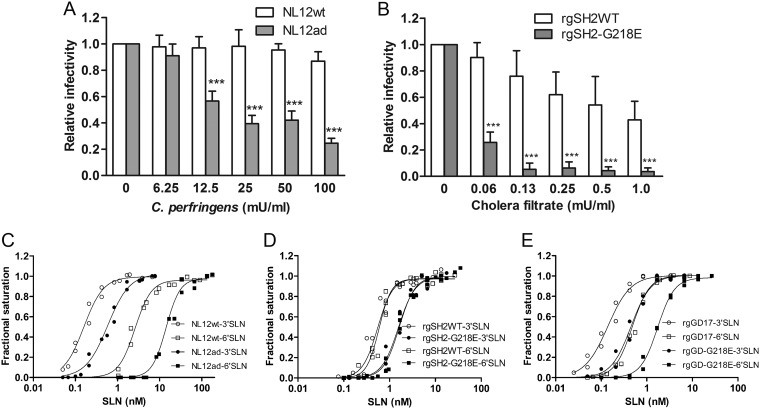
Virus binding to MDCK cells and receptor analogs. (A) Relative infectivity of NL12WT and NL12ad in the MDCK cells with reduced SA receptor density by C. perfringens pretreatment. (B) Relative infectivity of rgSH2WT and rgSH2-G218E in the MDCK cells with reduced SA receptor density by cholera filtrate pretreatment. Data (means ± the standard errors) were from four independent experiments. ***, *P* < 0.001 (two-way ANOVA with Bonferroni posttest). (C to E) BLI equilibrium binding to the immobilized 3′SLN or 6′SLN by NL12WT and NL12ad (C), rgSH2WT and rgSH2-G218E (D), and rgGD17 and rgGD-G218E (E). Each binding curve in panels C, D, and E was the best fit of data from two independent experiments to the “one site specific binding with Hill slope” equation determined using GraphPad Prism.

Because amino acid 218 is adjacent to the 220-loop (Fig. 2A), which is a critical component of RBS (15), the G218E substitution is likely relevant to alteration in virus binding to host cells. We first compared the virus binding avidity of NL12ad to MDCK cells to that of NL12WT. We reduced the sialic acid (SA) receptor density on the MDCK cell surface by pretreatment of cells with NA, followed by inoculation with 0.002 MOI of NL12WT or NL12ad for plaque formation and counting. As shown in Fig. 3A, the exogeneous NA resulted in concentration-dependent reduction of NL12ad infectivity but had an insignificant effect on NL12WT infectivity in the same dose. Given that the virus with a stronger receptor-binding avidity is more capable of binding to the cell surface with certain density of receptors, this result indicated a reduced receptor-binding avidity of the NL12ad virus.

The reduced binding to MDCK cells was also observed in rgSH2-G218E (Fig. 3B). Pretreatment of MDCK cells with cholera filtrate (receptor-destroying enzyme [RDE]) reduced the infectivity of both rgSH2WT and rgSH2-G218E in a concentration-dependent manner, but the latter showed a much steeper decline, suggesting that G218E substitution decreased the binding avidity of SH2 virus to MDCK cells.

### Reduced binding of G218E variants to both avian- and human-type receptor analogs.

We further assessed the binding of the MDCK-grown NL12, as well as egg-grown rgSH2 and rgGD17, to avian-type receptor (α2,3-linked SA) analogue 3′SLN and human-type receptor (α2,6-linked SA) analogue 6′SLN by using the biolayer interferometry (BLI) method. The trisaccharides 6′SLN and 3′SLN are commonly found at terminal positions of natural glycoconjugates, and 6′SLN is recognized by all human influenza A and B viruses (16). Simulating the MDCK cell binding experiment, we recorded the association of 1 mg/ml of each virus with different density of 3′SLN or 6′SLN immobilized on the biosensors. Analysis of the equilibrium virus-SLN binding demonstrated that the NL12WT binding to 3′SLN was 16-fold stronger than that to 6′SLN (the *K_d_* values were 0.16 nM 3′SLN and 2.6 nM 6′SLN), indicating an avian-type receptor preference. Without alteration in the receptor-binding preference, NL12ad bound to both 3′SLN and 6′SLN with nearly 20% of binding avidity of NL12WT (Fig. 3C). This finding is consistent with the reduced binding of NL12ad to MDCK cells.

However, the A(H7N9) CVV rgSH2WT bound almost equally to both 3′SLN and 6′SLN, with *K_d_*s of 0.55 nM for 3′SLN and 0.59 nM for 6′SLN, indicating that A(H7N9) has gained binding avidity to human-type receptor, but exhibited weaker binding to avian-type receptor than NL12WT. Similar to the finding with NL12ad, rgSH2-G218E variant exhibited a significantly decreased binding avidity to both 3′SLN and 6′SLN, which was about 35% of that measured from rgSH2WT (Fig. 3D). This finding is in agreement with the reduced binding of rgSH2-G218E to MDCK cells.

Interestingly, the BLI assay revealed the avian-type receptor preference of egg-grown rgGD17, with >2-fold stronger 3′SLN binding (*K_d_* of 0.14 nM) than the 6′SLN binding (*K_d_* of 0.48 nM). Compared to rgSH2WT, rgGD17 showed nearly 3-fold stronger binding to 3′SLN without undermined 6′SLN binding. Consistent with that observed in NL12ad and rgSH2-G218E, the binding avidity of rgGD-G218E to both avian- and human-type receptor analogs was reduced to approximately 25% of that measured in rgGD17 (Fig. 3E).

### G218E substitution facilitated N9 NA activity in H7N9 virus.

**FIG 4:**
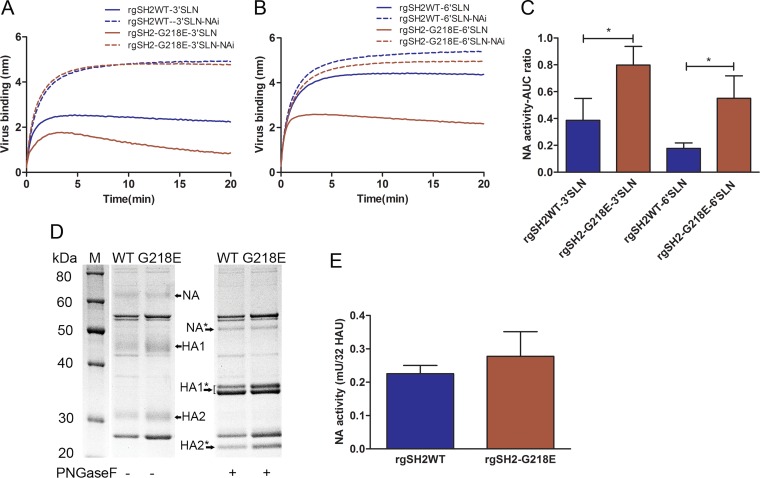
Evaluation of NA function. (A and B) Representative BLI recordings of the rgSH2 (blue) and rgSH2-G218E (red) binding with immobilized 3′SLN (A) and 6′SLN (B) in the presence of NAi (dashed line) or NAi-free (solid line) through the entire association phase. The difference between the dashed line and the solid line reflects the concurrent NA activity. (C) The (AUC_NAi_ – AUC_NAi-free_)/AUC_NAi_ ratio was calculated for comparison of the apparent NA activities of rgSH2WT and rgSH2-G218E (means ± the SD from three independent experiments). *, *P* < 0.05 (one-way ANOVA with Tukey posttest). (D) Separation of total viral proteins, with or without deglycosylation, from rgSH2WT and rgSH2-G218E in reduced SDS-PAGE gel (10% NuPAGE Bis-Tris gel, 5 μg of protein per sample, colloidal blue staining). The indicated HA and NA bands were confirmed by Western blotting (data not shown). *, Deglycosylated. (E) NA activities of rgSH2WT (blue) and rgSH2-G218E (red) measured by the 4-MU-NANA method. Means ± the SD from four samples are shown. *P* = 0.23 (*t* test).

To evaluate the functional interaction of HA and NA in the H7N9 viruses, we monitored the dynamic course of virus binding to the receptor analogs with simultaneous NA cleavage illustrated in the association phase of BLI kinetics recordings. To reveal the exclusive HA binding, we used NAi to inhibit enzymatic activity of NA. Figure 4A showed the saturation binding curves of both rgSH2WT and rgSH2-G218E binding to 3′SLN with NAi (dashed lines) or without NAi (solid lines). When the NA activity was inhibited by NAi, the association curves of rgSH2WT and rgSH2-G218E were similar: a rapid rise, followed by a gradual increase to reach the plateau, the equilibrium of association and dissociation between the virus and receptor analog. In contrast, the concurrent HA binding and NA cleavage yielded distinctive association curves in the absence of NAi. Even though the same amount of receptor analogs and virus were loaded, the peak of the rgSH2WT association curve was half of that in the presence of NAi. After a short ascending phase, the curve turned descending. The difference between the association curves with or without NAi revealed the disrupted virus-receptor binding by NA. Compared to the association curve of rgSH2WT with 3′SLN, the association curve of rgSH2-G218E to 3′SLN showed a lower peak, followed by a steeper decay in the absence of NAi, indicating a more efficient cleavage of the sialic acids by NA. Figure 4B demonstrated that the binding of rgSH2WT to 6′SLN was only slightly affected by the concurrent NA enzymatic activity (the difference between the blue solid line and blue dashed line), whereas binding of rgSH2-G218E to 6′SLN was more susceptible to the concurrent NA cleavage (red lines), indicating a stronger NA effect on binding to a human-type receptor analogs (6′SLN) of the rgSH2-G218E than that of the rgSH2WT.

The disrupted virus-receptor binding that resulted from the NA activity was quantitated by measuring the AUC difference in binding curves with or without NAi (Fig. 4C), which indicated a more efficient SA cleavage by the rgSH2-G218E than by the rgSH2WT, although the expression of NA on both rgSH2WT and rgSH2-G218E was comparable, as determined by SDS-PAGE (Fig. 4D), and the NA activities of rgSH2WT and rgSH2-G218E were similar in cleavage of the small soluble substrate 4-MU-NANA (Fig. 4E). Similar results were obtained in the study using rgGD17 and rgGD-G218E (data not shown). These data demonstrated that (i) the concurrent HA binding and NA cleavage dynamically shaped the distinctive interaction of H7N9 virus with avian- and human-type receptors, (ii) the N9 NA functioned more efficiently on disrupting the H7 virus binding to avian-type receptor than the binding to human-type receptor, and (iii) the G218E substitution in H7 HA resulted in an enhanced effect of N9 NA on disrupting virus binding to both avian- and human-type receptors as a result of reduced H7N9 virus binding.

### G218E substitution facilitated the release of progeny H7 viruses from infected MDCK cells.

**FIG 5:**
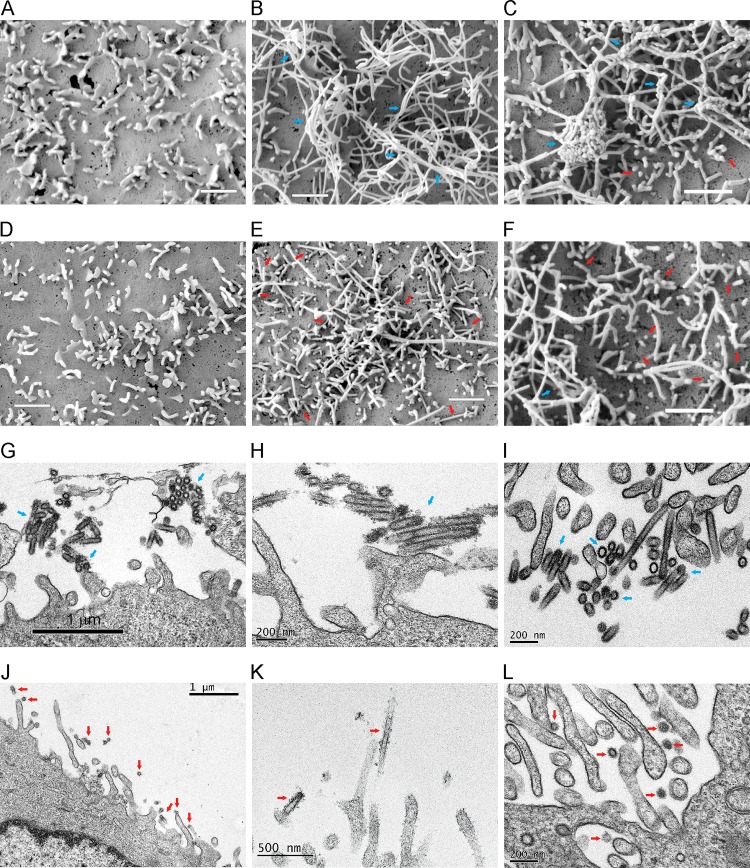
Release of nascent H7 viruses from MDCK cells. (A to F) Representative SEM images of MDCK monolayers after 2 and 10 h of infection with NL12WT (A and B) or NL12ad (D and E) and at 10 h postinfection with rgSH2WT (C) and rgSH2-G218E (F). Scale bars, 1 μm. (G to L) Representative TEM images of ultrathin sections from MDCK cells at 10 h postinoculation with NL12WT (G, H, and I) or NL12ad (J, K, and L). Blue arrows indicate the aggregated viruses on the surfaces of the infected cells; red arrows indicate individual virus budding and release.

Based on the findings that G218E resulted in reduced H7 virus binding to receptors and facilitated NA function, we further used electron microscopic techniques to determine whether G218E-induced MDCK adaptation is associated with facilitated nascent virus release from the infected cells. Under scanning electron microscopy (SEM), microvilli were the exclusive projections on MDCK cell surface at 2 h postinoculation (Fig. 5A and D). The filamentous NL12 virus budding was observed at 6 h postinoculation (data not shown). At 10 h postinoculation, a large number of tangled filamentous NL12WT virus particles were aggregated and detained on the surfaces of infected cells (Fig. 5B), while the clustered filaments were hardly observed in the NL12ad-infected MDCK cells (Fig. 5E). On the ultrathin sections examined by transmission electron microscopy (TEM), the typical findings were aggregated filaments attached to the NL12WT-infected cells (Fig. 5G to I) and the individual budding and release of filaments from NL12ad-infected cells (Fig. 5J to L).

Both spherical and filamentous morphologies were observed in MDCK-grown rgSH2WT and rgSH2-G218E. With SEM, we visualized numerous different-sized virus clusters on rgSH2-infected MDCK cells (Fig. 5C), but few aggregated viruses, among the individual viruses on the surfaces of rgSH2-G218E-infected cells (Fig. 5F). The SEM and TEM findings confirmed the impeded release of WT NL12 and SH2 progenies from infected cells, which indicated that insufficient NA function cooperates with the HA binding and that the G218E substitution facilitates nascent virus release to initiate a new replication cycle by reducing HA receptor binding.

## DISCUSSION

To optimize the growth of H7N9 CVV in MDCK cells and eggs, we introduced G218E, a previously selected H7 virus adaptation mutation, into the HAs of both SH2 and GD17 strains. As desirable as the phenotypic trait of improved replication in both MDCK cells and eggs, the antigenicity of both rgSH2-G218E and rgGD-G218E was comparable to that of the counterparts bearing G218, which was confirmed by two-way HI assay and MN assay, and highlighted by the effective protection of ferrets from challenge with the parental HPAI virus. Moreover, both rgGD17 and rgGD-G218E were tested nonpathogenic in egg embryos, chickens, or ferrets, and the modified sequence of the HA gene coding for the HA1/HA2 cleavage site remained unchanged after 10 passages in eggs (data not shown). To elucidate the mechanisms underlying the G218E phenotype, we further investigated the functional alteration related to the G218E substitution. The residue at position 218 of HA is at the interfaces of monomers, whereas G218E was previously reported to increase H3 HA membrane fusion pH that decreases HA acid stability (17). We therefore assessed the possible impact of G218E on HA stability in H7 viruses by a syncytium assay (18) and found no significant difference in HA fusion pH between NL12WT and NL12ad or between rgSH2 and rgSH2-G218E (data not shown), which indicated that G218E does not result in a significant change in H7 HA acid stability. Given that residue 218 is adjacent to the RBS, we investigated the relevance of the improved growth with the virus binding to host cells. Interestingly, our data demonstrated that G218E significantly reduced H7 receptor binding and apparently facilitated N9 function.

HA and NA are both glycoproteins embedded in influenza viral envelope and play critical but distinct roles in the virus replication cycle: HA initiates infection by binding to host cell SA receptors and docking fusion with endosome membrane to trigger replication, and NA sustains infection by cleaving SA from the host cell surface to release the nascent virions for new replication cycles and cleaving the SA from viral envelope to keep viruses from aggregation (19, 20). The functionally balanced HA receptor binding affinity and NA catalytic activity are essential for a productive influenza virus infection (21–23).

Adaptation of influenza virus from one host species to another engages genetic variation. Balancing the incompatible HA-NA function usually results from mutations in HA that alter the receptor binding affinity and in NA that change the catalytic function (24, 25). The single amino acid substitution Q226L in HA is known to change the HA receptor binding specificity from avian-type to human-type (26). In this study, we used the BLI method to measure the multivalent binding of whole virus to the immobilized SA receptor analogs. The results demonstrated that NL12WT and rgGD17 viruses, which possess Q226 in HA, preferentially bound avian-type receptor analog. In contrast, rgSH2WT with L226 showed increased human-type receptor binding but moderately reduced avian-type receptor binding compared to NL12WT and rgGD17, in agreement with the dual receptor specificity of the novel H7N9 virus reported by other investigators (27–33), although some investigators reported a more significant 2,3-SA preference of the H7 HA in receptor binding (34, 35), which might reflect a difference in experimental methods or materials (such as whole virus versus recombinant HA protein, or different receptor analogs). However, the NA-mediated dissociation was more appreciable on virus-3′SLN binding than on virus-6′SLN binding, which is consistent with previous findings (28, 34). The rgSH2WT efficiently attached the MDCK cells that bear both α2,3- and α2,6-SA (36, 37), but the progeny virus was retained on the infected cells, as shown in the SEM image, indicating insufficient NA function. G218E resulted in significant decrease in human- and avian-type receptor binding avidity and facilitated N9 NA activity, which apparently balanced H7 HA binding and N9 NA cleavage capacity, thus enabling the progeny to disseminate more efficiently. Located in the proximity of the RBS, the glycine at position 218 is highly conserved in H7 HA, and glutamic acid is the only substitution documented in less than 1% (13/1553) of H7 HA sequences deposited in NCBI GenBank. Without direct interaction with SA (38), the negatively charged glutamic acid probably causes the steric alterations of HA molecule and destabilization of interactions between SA and the residues within the RBS pocket, thereby weakening the strength of the binding.

In N9 NA, a second SA receptor binding site separated from the catalytic site was identified by X-ray crystallography (39). The second SA binding site is responsible for the NA hemadsorption activity (40) and likely enhances the H7N9 virus binding to human-type receptors (41). To address the role of the second SA binding site in the N9 NA of rgSH2 and rgSH2-G218E, we replaced the N9 NA with the N1 NA from PR8 to generate H7N1 reassortants. With the BLI method, we did not observe a significant difference in either the 3′SLN or the 6′SLN binding affinity between H7N9 and H7N1 reassortants, which indicated a minimal role of the second SA binding site of N9 NA on the binding of SH2 virus to receptor analogs (data not shown). This limited role of NA receptor binding could result from the T401A substitution of the second SA binding site in the NA from SH2 virus, which causes decreased N9 NA receptor binding, as well as reduced specific enzymatic activity (42).

Interestingly, the G218E substitution in HA was correlated with increased NA-mediated dissociation of the HA from the receptor analogs. Because no genotypic alteration was found in the NA of rgSH2-G218E and, moreover, neither the NA amount nor the enzymatic activity toward the soluble substrate MU-NANA was significantly different between rgSH2WT and rgSH2-G218E, the feasible rationale could be that G218E in HA increased SA accessibility for NA. The N9 NA from the novel A(H7N9) has a five-amino-acid deletion in its stalk in comparison to the most closely related avian N9 NA (43), which might compromise its receptor-destroying capacity by decreasing the accessibility to the cleavage site of the membrane-bound substrate (19). In addition, the T401A mutation could also contribute to the reduced NA function (42). The G218E substitution in HA possibly reduced the binding stability of the HA-receptor complexes, which made the SA more accessible for cleavage by viral NA. However, the precise mechanism remains to be elucidated. In summary, we have generated high-growth H7N9 CVVs by introduction of a single amino acid substitution (G218E) in HA, which increased virus replication by balancing HA-NA functions. Ferrets immunized with rgGD-G218E or rgGD17 were protected from HPAI GD17WT challenge, suggesting that the G218E substitution does not alter the HA antigenicity of the GD17 virus. With the high yield in cells and eggs, the expected antigenic properties, as well as the low-pathogenic characteristics in eggs and animals, H7N9 CVV with G218E mutation, recently designated CBER-RG7D (https://www.who.int/influenza/vaccines/virus/en/), is anticipated to facilitate the pandemic preparedness against the A(H7N9) influenza threat.

## MATERIALS AND METHODS

### Cells and viruses.

We used MDCK cells (American Type Culture Collection, Manassas, VA) for virus growth and Vero cells (BioReliance; Invitrogen) for CVV preparation. MDCK cells were maintained in Eagle minimal essential medium (EMEM; Lonza, Basel, Switzerland) supplemented with 5% fetal bovine serum (FBS; HyClone; Thermo Scientific). Vero Working Cell Bank was thawed and maintained in Dulbecco modified Eagle medium (Gibco, Thermo Scientific, USA) supplemented with 10% FBS. Both types of cells were incubated at 37°C in a humidified atmosphere containing 5% CO_2_. For virus propagation in eggs, 10-day-old specific-pathogen-free chicken embryonated eggs (Charles River Laboratories, Malvern, MA) were used.

The viruses used in this study are listed in Table 2. Briefly, wild-type viruses were received from the indicated providers. The MDCK-adapted progeny (NL12ad) was selected through three passages in MDCK cells. The reassortant viruses were generated by the reverse genetics method (44) with the HA and NA genes of interest cotransfected with six other gene segments from the donor virus A/Puerto Rico/8/1934 (PR8) in Vero cells. The designated mutations were generated by using a QuikChange Lightning site-directed mutagenesis kit (Agilent Technologies, Santa Clara, CA). For rgSH2, the HA (GISAID EpiFlu, EPI439502) and NA (GISAID EpiFlu, EPI439500) genes were synthesized (GenScript USA, Inc., Piscataway, NJ). For rgGD17, the HA and NA genes from the highly pathogenic A/Guangdong/17SF003/2016 (GD17WT) were initially PCR amplified and cloned into cloning vector pGEM-T Easy (Promega, Madison, WI) for modification, with which we deleted the codon for the multibasic cleavage site RKRT from the HA gene and generated K292R (N2 numbering) mutation in NA to restore the neuraminidase inhibitor (NAi) sensitivity. The modified HA and NA genes were then cloned into the pHW2000 vector (St. Jude Children’s Research Hospital, Memphis, TN) to generate the low-pathogenic, NAi-sensitive rgGD17 reassortant virus. The rescued viruses were propagated in either MDCK cells or eggs, and the infectivity was determined by both plaque assay in MDCK cells and from the 50% egg infective dose in eggs. All of the reassortant A(H7N9) viruses were rescued and maintained in the biosafety level 3 (BSL-3) facility.

**TABLE 2:** Viruses generated and analyzed in this study

Strain	Subtype	Genotype	Abbreviation
A/mallard/Netherlands/12/2000 (U.S. CDC)	H7N3	Wild type	NL12WT
NL12-MDCK adapted	H7N3	HA: G218E, K328R (H3#)	NL12ad
rg^*a*^NL12WT (7:1)	H7N1	7:1^*b*^, NL12 HA-WT	rgNL12WT
rgNL12-G218E (7:1)	H7N1	7:1, NL12 HA-G218E	rgNL12-G218E
rgNL12-K328R (7:1)	H7N1	7:1, NL12 HA-K328R	rgNL12-K328R
rgNL12-G218E/K328R (7:1)	H7N1	7:1, NL12 HA-G218E/K328R	rgNL12-G218E/K328R
rgA/Shanghai/2/2013	H7N9	6:2^*c*^, SH2 HA-WT	rgSH2WT
rgA/Shanghai/2/2013-G218E	H7N9	6:2, SH2 HA-G218E	rgSH2-G218E
rgSH2WT (7:1)	H7N1	7:1, SH2 HA-WT	rgSH2WT (H7N1)
rgSH2-G218E (7:1)	H7N1	7:1, SH2 HA-G218E	rgSH2-G218E (H7N1)
A/Guangdong/17SF003/2016 (HPAI) (China CDC)	H7N9	Wild type	GD17WT
rgA/Guangdong/17SF003/2016 (LPAI)	H7N9	6:2, GD17 HA_ΔRKRT_	rgGD17
rgA/Guangdong/17SF003/2016-G218E	H7N9	6:2, GD17 HA_ΔRKRT_-G218E	rgGD-G218E

a Reverse genetics.

b 7:1 reassortant: HA from the parental virus, and the rest 7 gene segments from donor virus PR8.

c 6:2 reassortant: HA and NA from the parental virus, and the rest 6 gene segments from PR8.

### Growth kinetics.

Virus replication kinetics was evaluated with multistep growth curve. Briefly, confluent monolayers of MDCK cells grown in 12-well plate were inoculated with virus at an MOI of 0.01 for 1 h, followed by washing and then incubation with UltraMDCK serum-free culture medium (Lonza, Basel, Switzerland) containing TPCK-trypsin (1.0 μg/ml; Sigma, St. Louis, MO) at 33°C for 72 h. An aliquot of culture medium from each well of infected cells was collected at 8, 24, 48, and 72 h after infection and stored at –80°C for titration by plaque assay.

### Virus purification.

For propagation of viruses in MDCK cells, confluent MDCK monolayers in six T-75 Corning cell culture flasks were inoculated with virus (MOI of 0.01) and incubated in UltraMDCK serum-free medium containing 1 μg/ml of TPCK-trypsin at 35°C for 72 h. The collected MDCK culture media were clarified by centrifugation (2,000 × *g*, 20 min, 4°C), and the final volume of the clarified culture medium for each virus preparation was 90 ml.

Each egg-grown virus stock was prepared by propagation of the virus in fifteen 10-day-old eggs at 35°C for 72 h. The harvested allantoic fluid was clarified by centrifugation (2,000 × *g*, 20 min at 4°C), and the final volume of the clarified allantoic fluid used for virus purification was ≥120 ml.

The MDCK-grown or egg-grown virus was pelleted by ultracentrifugation using a Beckman type 45 Ti rotor (104,350 × *g*, 90 min, 4°C), resuspended in phosphate-buffered saline (PBS; pH 7.2), and purified through a 30 to 60% sucrose gradient (106,500 × *g*, 4°C for 90 min) using a Beckman SW 32 Ti rotor and 17-ml tubes. The virus-containing fraction was collected and diluted in 15 ml of PBS, pelleted by ultracentrifugation (106,500 × *g* for 60 min at 4°C) using a Beckman SW 32 Ti rotor and 17-ml tubes, and resuspended in PBS. The protein content of the purified virus was determined by using a Pierce Micro BCA protein assay kit (Thermo Fisher Scientific, Waltham, MA).

### Ferret study.

The ferret study was performed in a BSL-3 containment laboratory according to protocols approved by the FDA/Center for Biologics Evaluation and Research (CBER) Animal Care and Use Committee. Male ferrets, 3 to 4 months of age, were purchased from Triple F Farms (Gillett, PA) and prescreened to confirm they were seronegative to the currently circulating seasonal influenza strains or A(H7N9) viruses. Each ferret was subcutaneously implanted with an IPTT transponder for identification and temperature monitoring using a DAS-7007 R reader system (BioMedic Data Systems, Inc., Seaford, DE). For the immunogenicity study, each ferret was intranasally (i.n.) inoculated with two doses (8.7 × 10^6^ PFU) of specific CVV administered 2 weeks apart. Blood for the serum preparation was collected at 4 weeks after the initial inoculation. For the HPAI A(H7N9) challenge study, nine ferrets were randomly assigned into three groups: (i) naive ferrets; (ii) rgGD17-immunized ferrets, where each ferret was inoculated i.n. with 1.0 ml of rgGD17 (8.7 × 10^6^ PFU); and (iii) rgGD17-G218E-immunized ferrets, where each ferret was inoculated i.n. with 1.0 ml of rgGD17-G218E (8.7 × 10^6^ PFU), followed by monitoring of temperature and body weight for 2 weeks. After venous blood sample collection, as well as the baseline body weight and temperature recording, at day 14 postimmunization, each ferret was challenged i.n. with 1 ml of HPAI GD17WT (5 × 10^7^ PFU) and placed under close observation for body weight, temperature, and signs of illness for 2 weeks. A ferret would be euthanized upon more than 20% body weight loss or the occurrence of signs of a central nervous system disorder, e.g., abnormal movement and opisthotonus. The procedures used for i.n. inoculation, venous blood collection, and nasal washing were performed under light sedation using ketamine and xylazine. Blood samples were collected for antibody titration from all surviving animals on day 15 postchallenge.

### Hemagglutination inhibition assay.

The antigenicity of the virus was evaluated by HI assay (45). Sera from ferrets immunized with A(H7N9) viruses were pretreated with receptor-destroying enzyme (RDE; Denka Seiken Co., Ltd., Japan) and serially diluted in 2-fold increments. Then, 25-μl portions of the diluted serum sample were mixed with 25 μl of virus (4 HA units) in 96-well plates, incubated at room temperature for 30 min, and then incubated with 50 μl of 1% horse red blood cells for 1 h at room temperature. The HI titer was determined as the reciprocal of the highest serum dilution at which the agglutination was inhibited. The detection limit of this assay was a titer of 10.

### Microneutralization assay.

Microneutralization assay (46) was performed to evaluate the specific neutralizing antibody in the immunized ferret sera. The RDE-treated ferret sera were serially diluted with serum-free EMEM from columns 1 to 11 in a 96-well transfer plate at 25 μl/well, followed by the addition of 100 50% tissue culture infective doses (TCID_50_) of the tested virus (100 TCID_50_/25 μl), and then incubated at room temperature for 1 h. The virus-serum mixture was then transferred to the MDCK monolayer grown in a 96-well tissue culture plate for virus adsorption with column 12 as a negative control. After a 20-h inoculation at 37°C, the inoculum was removed and replaced with 100 μl of serum-free EMEM containing TPCK-trypsin (1 μg/ml) and then incubated at 33°C for 5 days. Subsequent to crystal violet staining, the cytopathic effect (CPE) on MDCK monolayers was examined, and the neutralizing antibody titer was determined as the reciprocal of the highest serum dilution at which no CPE was detected. The detection limit of this assay was a titer of 10.

### Virus binding avidity to MDCK cells.

To understand whether the MDCK adaptation was related to a change in virus binding to host cell receptors, we assessed the virus binding avidity to MDCK cells with different sialic acid (SA) densities resulting from treatment by exogenous NA. Confluent MDCK monolayer cells grown in 12-well plates were preincubated with a serial dilution (6.25 to 100 mU/ml) of NA from Clostridium perfringens (Roche, catalog no. 11585886001 [purchased from Sigma, St. Louis, MO]) for 1 h in the presence of 2 mM Ca^2+^ at 37°C, followed by a thorough PBS washing, and then inoculated with NL12WT and NL12ad viruses (MOI of 0.002) for 1 h at 37°C or preincubated with a serial dilution (0.06 to 1.0 mU/ml) of cholera filtrate NA (catalog no. 8772; Sigma, St. Louis, MO) in PBS containing 5 mM Ca^2+^ for 1 h, followed by washing with PBS, and subsequently inoculated with rgSH2WT or rgSH2-G218E (MOI of 0.002) for 1 h at 37°C. After removal of the inoculum and wash, the cells were covered with 0.8% agarose overlay containing 1 μg/ml of TPCK-trypsin and incubated at 33°C in an atmosphere with 5% CO_2_ for 72 h. The virus infectivity ratio was calculated by normalizing the plaque counts of the NA-pretreated wells to those from the wells without NA pretreatment.

### Biolayer interferometry.

Purified whole virus binding to immobilized α2,3- and α2,6-SA receptor analogs was measured with an Octet QK^e^ biolayer interferometer using streptavidin biosensors (Pall ForteBio Corp., Menlo Park, CA) (34). Avian-type receptor analog Neu5Acα2-3Galβ1-4GlcNAcβ-PAA-biotin (3′SLN, catalog no. 01-077) and human-type receptor analog Neu5Acα2-6Galβ1-4GlcNAcβ-PAA-biotin (6′SLN, catalog no. 01-130) were purchased from GlycoTech Corp. (Gaithersburg, MD). The 3′SLN and 6′SLN are polyacrylamide polymers of ∼30 kDa containing 5% mol biotin and 20% mol carbohydrate.

The biotinylated SLN polymers were serially diluted in binding buffer (NaCl, 150 mM; HEPES, 10 mM; EDTA, 3 mM; 0.005% Tween 20) ranging from 0.05 to 26.7 nM for 3′SLN and 0.1 to 133 nM for 6′SLN and then immobilized on the streptavidin biosensors for 10 min, followed by virus (1.0 mg/ml) association at 25°C for 30 min and subsequent dissociation in virus-free buffer containing calcium. In the preliminary experiments, we confirmed that the NAs from all the tested viruses, including NL12 (N3), SH2 (N9), and rgGD17 (N9), were sensitive to NAi zanamivir hydrate (M1826; Moravek Biochemicals, Inc., Brea, CA). To minimize the NA-induced virus dissociation from the immobilized 3′SLN or 6′SLN, the entire association phase was recorded in the presence of 10 μM zanamivir.

For steady-state analysis, the equilibrium response, i.e., the plateau amplitude of virus bound to the immobilized 3′SLN or 6′SLN at the respective dilution, was measured at the end of a 30-min association phase. Data from two independent experiments using viruses from different preparations were analyzed by GraphPad Prism 5.0 software (GraphPad Software, Inc., La Jolla, CA), best fit to the following equation:

$$Y=B_{\max}\,\times [SLN{]}^{h}/\left(K_{d}^{h}+[SLN{]}^{h}\right)$$

where *Y* is the specific binding, *B*_max_ is the maximal specific binding at equilibrium (measured as nm), *K_d_* is the SLN concentration (nM) needed to achieve a half-maximum binding at equilibrium, corresponding to the equilibrium binding constant, and *h* is the Hill slope, reflecting the binding cooperativity (*h* = 1, noncooperative binding; *h* > 1, positively cooperative binding; *h* < 1, negatively cooperative binding).

To evaluate the cohabited NA function, we measured the destroyed virus-receptor analog binding by using NAi. The 20-min associations of the same virus with 3′SLN and 6′SLN were simultaneously recorded in both NAi-containing buffer and NAi-free buffer containing 5 mM CaCl_2_, and the area under the curve (AUC) for each binding curve was quantitated by using GraphPad software. Because the difference between the AUC of NAi-containing and that of NAi-free reflected the NA-destroyed binding, the apparent NA function is presented as the following AUC ratio: (AUC_NAi-containing_ – AUC_NAi-free_)/AUC_NAi-containing_.

### NA activity assay.

NA activity of the SH2 viruses was evaluated by the MU-NANA assay as previously described (47). Briefly, 50 μl of 20 μM 4-methylumbelliferyl-*N*-acetylneuraminic acid (MU-NANA; Sigma, St. Louis, MO) was added to the same volume of sample containing 32 HAU virus to incubate for 1 h at 37°C. The control (PBS [pH 7.4]) and a serially diluted standard (NA from *Vibrio cholerae*; Sigma, N7885) were incubated in parallel. Reactions were stopped with 0.1 M glycine (pH 10.7) in 25% ethanol, and the fluorescence was measured on an automated plate reader Victor V (Perkin-Elmer, Waltham, MA) at an excitation of 355 and an emission of 460 nm for 0.1 s per well. The viral NA activity was calculated using the standard curve of the *Vibrio cholerae* NA.

### Scanning electronic microscopy.

SEM was used to visualize virus release from the host cells. MDCK cells were grown on 12-mm poly-l-lysine-coated circular glass coverslips (Neuvitro, Vancouver, WA) in a 12-well plate at one slide per well. Cells on five slides were inoculated with the indicated virus at an MOI of 3 and then incubated at 37°C. One slide from each viral infection was washed and fixed with 2.5% glutaraldehyde at 2, 6, 8, 10, or 12 h postinfection, respectively, and postfixed with 1% OsO_4_. Cells were then dehydrated and critical point dried. The slides were mounted on tubs and coated with gold-palladium for examination using MIRA3 SEM (Tescan, Brno, Czech Republic).

### Transmission electron microscopy.

MDCK cells (5 × 10^5^) were seeded on a preincubated Corning Transwell permeable support (polycarbonate membrane, 3.0-μm pore size; Costar, catalog no. 3402) inserted in a 12-well plate and incubated at 37°C for 16 h. After inoculation with virus (MOI of 3) from the apical side at 37°C for 2 h, the cells were washed and then incubated in UltraMDCK medium (without TPCK-trypsin) at 33°C. At 10 h postinfection, the cells were washed with PBS, fixed in 2.5% glutaraldehyde, and postfixed with 1% OsO_4_. After dehydration, the polycarbonate filter was embedded in Epon12 epoxy resin at 60°C for 48 h. The samples were then sectioned, stained with uranyl acetate and lead citrate, and examined with a Zeiss Libra 120 Plus transmission electron microscope. Images were taken with a Gatan US100XP digital camera.

### Statistical analysis.

The quantitative experiments were repeated at least three times, and results were analyzed with a *t* test (two tails), a one-way analysis of variance (ANOVA) with *post hoc* Tukey test, or a two-way ANOVA with the Bonferroni posttest using GraphPad Prism 5.0 software. A *P* value of <0.05 was considered statistically significant.
